# HEALTH AFTER AGE 50: THE ROLE OF ADULT CHILDREN’S EDUCATION FOR OLDER PARENTS’ FRAILTY IN EUROPE

**DOI:** 10.1093/geroni/igad104.1741

**Published:** 2023-12-21

## Abstract

Social stratification research has shown that family background can significantly impact a child’s life chances. However, intergenerational transmission of (dis-)advantages is a two-way process, because parental investment in children may be reciprocated later in life. Particularly, very little is known about the importance of adult children’s socio-economic resources on ageing parents’ well-being – what has been called the effect of the “social foreground” on later-life health. This study aims to investigate whether and how adult offspring’s educational attainments are associated with parents’ health. This study uses dyadic panel data from all the available waves of the Survey of Health, Ageing and Retirement in Europe (SHARE) (i.e., from 2004 to 2020). The sample is composed of 89,752 parents (210,999 parent-child dyads, 250,605 observations) aged 50 or older living in 29 different European countries. Our preliminary results from random intercept linear regression models show that child education is negatively associated with the 40-item Frailty Index (FI) among parents. This association is particularly strong among lower-educated parents (especially mothers). These findings highlight the importance of considering the socio-economic resources of adult children when examining the health and well-being of older adults. This suggests that public investments in younger generations’ education may benefit older generations as well. In developing future analyses, we plan to focus on identifying the potential generative mechanisms behind this relationship and exploring potential policy interventions to support ageing parents and their families.

